# Phosphorylation-driven effector switching of Rab7 and Rab12 by the leucine-rich repeat kinase 1 in mast cells

**DOI:** 10.3389/fimmu.2025.1709196

**Published:** 2025-11-21

**Authors:** Jana Omar-Kabha, Sewar Omari, Yaara Gorzalczany, Fatima Amer-Sarsour, Elisabeth Kleeblatt, Mitsunori Fukuda, Avraham Ashkenazi, Ronit Sagi-Eisenberg

**Affiliations:** 1 Department of Cellular, Developmental, and Regenerative Biology, Gray Faculty of Medical & Health Sciences, Tel Aviv University, Tel Aviv, Israel; 2 Laboratory of Membrane Trafficking Mechanisms, Department of Integrative Life Sciences, Graduate School of Life Sciences, Tohoku University, Sendai, Miyagi, Japan; 3 Sagol School of Neuroscience, Tel Aviv University, Tel Aviv, Israel

**Keywords:** mast cells, IgE, MRGPRX2, Rab7, Rab12, protein kinase C, LRRK1, LRRK2

## Abstract

**Introduction:**

Mast cells (MCs) mediate immune, allergic, and neuroinflammatory responses by releasing inflammatory mediators upon activation through the immunoglobulin E (IgE) receptor FcεRI or innate stimuli acting through Mas-related G protein coupled receptors (Mrgprs). We previously showed that Rab12 negatively regulates mediator release by recruiting the Rab-interacting lysosomal protein (RILP)-dynein complex to the secretory granules (SGs). Because Rab12 also interacts with the RILP-like proteins RILP-L1 and RILP-L2, we examined whether phosphorylation controls Rab12 distribution among its RILP family effectors.

**Methods:**

Pulldown assays were used to compare the effects of MC activation on Rab12 interactions with its effectors.

**Results:**

Here we show that activation by either IgE/antigen or the neuropeptide substance P, which binds to MRGPRX2, induces phosphorylation of the Rab GTPases Rab7 and Rab12. Phosphorylation of both GTPases was sensitive to protein kinase C (PKC) inhibition but resistant to inhibition of the leucine-rich repeat kinase 2 (LRRK2), a known Rab12 phosphorylating kinase. Furthermore, knockdown of the Leucine-Rich Repeat kinase 1 (LRRK1) suppressed phosphorylation of both Rab7 and Rab12, implicating LRRK1 in their phosphorylation by a PKC-dependent mechanism. Like phosphorylation by LRRK2, LRRK1-mediated phosphorylation of Rab12 increased its affinity for RILP-L1 and RILP-L2 while reducing binding to RILP. In contrast, LRRK1 phosphorylation of Rab7 enhanced its affinity for RILP.

## 1 Introduction

Mast cells (MCs) are key regulatory cells of the immune system that play important roles in innate defense against infection (1–4) and envenomation (5), but also contribute to allergy and anaphylaxis (6), as well as inflammation linked with autoimmunity, cancer, neuroinflammation and neurodegenerative diseases (7–10). Both the physiological and pathophysiological functions of MCs in health and disease are primarily mediated by the release of a variety of inflammatory mediators (11), some of which, such as histamine, are pre-formed and stored in lysosome-related secretory granules (SGs) (12, 13). These SGs release their contents through regulated exocytosis in response to diverse adaptive and innate triggers (1, 3, 14, 15). The adaptive trigger involves the binding of allergen-specific Immunoglobulin E (IgE) to FcϵRI, the high-affinity receptor for IgE, followed by crosslinking upon allergen encounter (14, 16). Innate triggers activate MCs independently of IgE and include antimicrobial peptides and neuropeptides such as substance P, which act through Mas-related G protein-coupled receptors (Mrgprs) expressed by a subset of MCs, including connective tissue MCs in rodents (17) and skin, fat, colon and synovial MCs in humans (18, 19).Through their interaction with Mrgprs, and in particular with MRGPRX2, the human member of this receptor family, neuropeptides induce neurogenic inflammation (20), while certain drugs evoke pseudo-allergy (17).

We have previously shown that secretion triggered by FcϵRI, MRGPRX2 or by the combination of Ca^2+^ ionophore and phorbol ester, which activate MCs downstream of receptor signalling by elevating cytosolic Ca^2+^ and activating protein kinase C (PKC), is inhibited by expression of a constitutively active (CA) Rab12 mutant (21). Conversely, Rab12 knockdown potentiated secretion (22), indicating that Rab12 acts as a negative regulator of MC secretion downstream of receptor signalling, consistent with the established role of Rab GTPases as master regulators of intracellular trafficking (23). Rab GTPases exert their functions through GTP-dependent interactions with their effector proteins. Active Rab12 binds all three members of the RILP family, RILP, RILP-Like 1 (RILP-L1) and RILP-Like 2 (RILP-L2) (22, 24–26). Among these effectors, only RILP mediates Rab12-regulated recruitment of dynein, a minus-end motor, to SGs, thereby driving their perinuclear accumulation (22). This unique function of the Rab12–RILP complex prompted us to ask whether the distribution of Rab12 among its effectors is subject to regulation. Rab12 has recently gained attention as one of the physiological substrates of the Leucine-Rich Repeat kinase 2 (LRRK2) (27), a kinase whose increased activity occurs in idiopathic Parkinson’s disease (PD) and mutations leading to increased kinase activity comprise the most common cause of familial PD (28). Furthermore, phosphorylation by LRRK2 increases Rab12 affinity for RILP-L1 and RILP-L2 (25, 27, 29). Given the wide range role of LRRK2 in controlling organelle dynamics (30), we hypothesized that Rab12 might undergo phosphorylation in MCs and this phosphorylation may serve as a switch, to differentially tune Rab12 binding preferences within the RILP family. Surprisingly, we found that Rab12 and also Rab7 undergo phosphorylation in activated MCs. However, this phosphorylation involves the LRRK2 homolog kinase LRRK1. Furthermore, phosphorylation by either LRRK2 or LRRK1 exerts distinct effects on Rab12 versus Rab7 effector interactions, revealing phosphorylation as a key regulatory mechanism that fine-tunes their functions.

## 2 Materials and methods

### 2.1 Antibodies and reagents

Rabbit polyclonal anti-Rab12 [dilution 1:1000] (cat #18843-1-AP) was from Proteintech, (Rosemont, IL, USA). Rabbit monoclonal anti-Rab12 (phospho S106) [dilution 1:1000] (cat #ab256487), anti-LRRK1 [dilution 1:1000] (cat #ab228666) and Rabbit monoclonal anti-Rab7 (phospho S72) [dilution 1:1000] (cat #ab302494) were from Abcam (Cambridge, UK). Rabbit polyclonal Mouse monoclonal anti-Rab7 [dilution 1:1000] (cat #sc-376362) and Mouse monoclonal anti-GAPDH [dilution 1:10,000] (cat #sc-365062) were from Santa-Cruz Biotechnology (Dallas, TX, CA, USA). Horseradish-peroxidase (HRP)–conjugated goat anti–rabbit IgG [dilution 1:10,000] (cat #111-035-003) and Horseradish-peroxidase (HRP)–conjugated goat anti–mouse IgG [dilution 1:10,000] (cat #115-035-166) were from Jackson ImmunoResearch Laboratories (West Grove, PA, USA). Monoclonal anti-T7 IgG (Cat #69522-3) was from Novagen. Hilyte Plus 647-conjugated goat anti-mouse IgG (Cat #AS-61057-05-H647) was from Anaspec (Fremont, CA). DNP-specific IgE was derived from myeloma IgE producing cells (clone Hi 26.86), a kind gift from Dr. Ulrich Blank (Inserm, Paris, France). Human myeloma IgE (cat #401152), Rabbit anti human IgE- clone RM122 (cat # 04-1649), DNP-HSA [Ag]cat #A6661) and calcium ionophore A23187 [Ion] (cat #C7522), were from Sigma-Aldrich Chemicals Co (St. Louis, MO, USA).12-O-tetradecanoylphorbol-13-acetate [TPA] (cat #524400) was from Calbiochem (San Diego, CA, USA). GSK2578215A (cat #4629) and GF109203X (cat #0741) were from Tocris Bioscience (Bristol, UK). Go6976 (cat #G-1017) was from A.G. Scientific (San Diego, CA, USA). LY333531 (cat #13964) was from Cayman Chemical (Ann Arbor, MI, USA). MLi-2 (cat #HY-100411) was from MedChemExpress (MCE), (Monmouth Junction, NJ, USA). ON-TARGETplus SMARTpool human LRRK1 siRNA (cat #L-005320-00-0005) and non-targeting control pool siRNA (cat #D-001810-10-05) were from Horizon Discovery (Waterbeach, UK).

### 2.2 Plasmids used in this study

The following expression plasmids were used in this study: cDNAs of mouse RILP, RILP-L1, and RILP-L2, subcloned into the pGEX-4T-3 vector (GE Healthcare, Chicago, IL) and pEF-T7-RILP were prepared as described in (31). pEGFP-C1-Rab12 was prepared as described in (32).

Neuropeptide Y (NPY) fused to monomeric red fluorescence protein (NPY-mRFP) was a kind gift from Dr. U. Ashery (Tel-Aviv University, Tel Aviv, Israel).

### 2.3 Cell culture

RBL cells (the RBL-2H3 subline) were maintained as adherent cultures in low glucose DMEM (cat #01-050-1A), Biological Industries (Sartorius; Gottingen, Germany), supplemented with 10% FBS (cat #10270106), Gibco (Grand Island, NY, USA), 2 mM L-glutamine (cat #03-020-1A) Biological Industries (Sartorius; Gottingen, Germany), 100 μg/ml streptomycin and 100 units/ml penicillin (cat #03-031-5C) Biological Industries (Sartorius; Gottingen, Germany). RAW264.7 cells were maintained as adherent cultures in high glucose DMEM, supplemented with 10% FBS, 2 mM L-glutamine, 100 μg/ml streptomycin and 100 units/ml penicillin. LAD-2 cells (a kind gift from Dr. A.S. Kirshenbaum and Dr. D.D. Metcalfe, Laboratory of Allergic Diseases, National Institute of Allergy and Infectious Diseases, National Institutes of Health, Bethesda, MD) were cultured in StemPro (cat #10641-025) Gibco (Grand Island, NY, USA), supplemented with 100 ng/ml hrSCF (cat #300-07), Pepro-tech (Rocky Hill, NG, USA), 2 mM glutamine, 100 μg/ml streptomycin and 100 units/ml penicillin. All cells were maintained in a humidified incubator with 5% CO_2_ at 37°C. Mouse bone marrow derived MCs (BMMCs) were cultured as previously described (33). Briefly, bone marrows were obtained from hips, femurs, and tibias of 8- to 10-week-old female C57BL/6 mice and cells were cultivated in complete medium consisting of RPMI-1640 (cat # R8758, Sigma Aldrich) supplemented with 10% FBS, 2 mM L- glutamine, 100 μg/ml streptomycin, 100 units/ml penicillin, 12.5 units/ml Nystatin (cat # 03-032-1B, Biological Industries), 1 mM sodium pyruvate (cat # 03-042-1B, Biological Industries), 10 mM HEPES (pH 7.4) (cat # 03-025-1B, Biological Industries), 57.2 nM 2-mercaptoethanol (cat # M6260, Sigma Aldrich) and in the presence of 20 ng/ml IL-3 (cat # 213-13, Peprotec, Rocky Hill, NJ, USA). Cells were maintained in a humidified incubator with 5% CO_2_ at 37°C. MC purity was confirmed after about 8 weeks in culture by quantifying the percentage of cells expressing cKIT and FcϵRI by flow cytometric analysis (cell purity 90-95%).

### 2.4 MC activation

For IgE-mediated activation, RBL cells (8 X 10^6^ cells/plate) were grown overnight in the presence of a 1:512 dilution of conditioned media derived from a DNP specific IgE secreting Hybridoma and LAD-2 cells (0.5 X 10^6^ cells/ml) were grown overnight in the presence of 0.5 μg/ml of human myeloma IgE. After three washes in Tyrode’s buffer (20 mM HEPES pH 7.4, 137 mM NaCl, 2.7 mM KCl, 1.8 mM CaCl_2_, 1 mM MgCl_2_, 0.4 mM NaH_2_PO_4_, 5.6 mM Glucose and 0.1% BSA), cells were either left untreated or incubated for the desired time with 50 ng/ml of DNP-HSA (Ag) (RBL cells) or 10 μg/ml Rabbit anti human IgE (LAD-2 cells). For IgE-independent activation, RBL cells (8 X 10^6^ cells/plate), BMMCs (1 X 10^6^ cells/ml) and LAD-2 cells (0.5 X 10^6^ cells/ml) were washed in Tyrode’s buffer and either left untreated or triggered with a combination of 1 μM Ca^2+^ ionophore A23187 (Ion) and 50 nM TPA, or with 10 μM of substance P, for the desired time periods. For inhibitor treatments, cells were incubated for either 12 hours with 10 μM of GSK2578215A or for 2 hours with 100 nM of MLi-2, or for 30 minutes with either 1 μM of Go6976, 1 μM of GF109203X or 1 μM of LY333531, prior to cell trigger.

### 2.5 Macrophage activation

RAW264.7 cells (8 × 10^6^ cells/plate) were either left untreated or incubated with 10 μM of GSK2578215A for 12 hours at 37°C. Untreated cells were then incubated with either vehicle (0.05% DMSO) or with 100 nM of MLi-2 for 1.5 hours or 1 μM of LY333531 for 30 minutes, at 37°C, prior to cell trigger with a combination of 1 μM Ca^2+^ ionophore A23187 (Ion) and 50 nM TPA for 30 minutes at 37°C.

### 2.6 Pulldown assays

Pulldown assays were performed as previously described (22). Briefly, 20 μg of GST fusion proteins or control GST immobilized on Glutathione Agarose beads (cat #G4510, Sigma Aldrich Chemicals Co) were incubated for 18 hours at 4 °C with 500 μg of cell lysates prepared in lysis buffer comprising: 50 mM HEPES pH 7.4, 150 mM NaCl, 1 mM MgCl_2_, 1% TritonX100 (cat #T8787, Sigma Aldrich Chemicals Co), 1 mM Phenylmethanesulfonyl fluoride [PMSF] (cat #P7626, Sigma Aldrich Chemicals Co), cOmplete™ EDTA-free protease inhibitor cocktail (cat # 11873580001, Roche, Basel, Switzerland), 2 mM Sodium orthovanadate [Na_3_VO_4_] (cat #S6508, Sigma Aldrich Chemicals Co), 10 mM Sodium pyrophosphate tetrabasic decahydrate [NaPPi] (cat #S6422, Sigma Aldrich Chemicals Co) and 80 mM β-glycerophosphate (cat #14405, Cayman Chemical Company. At the end of the incubation period, beads were sedimented by centrifugation at 5000 x g for 5 minutes at 4°C, washed 4 times with the same buffer, supplemented with 0.2% TritonX100, and finally suspended in sample buffer. Samples were boiled for 7 minutes and subjected to SDS-PAGE and immunoblotting.

### 2.7 Western blot analysis

Cell lysates were separated by SDS-PAGE using 12-15% polyacrylamide gels for the detection of Rab7 or Rab12, or 7.5% polyacrylamide gels for the detection of LRRK1, and electrophoretically transferred to nitrocellulose membranes. Blots were blocked for 20 minutes in TBST (10 mM Tris-HCl pH 8.0, 150 mM NaCl, and 0.05% Tween 20 (cat #P1379, Sigma Aldrich Chemicals Co) containing 5% DifcoTM Skim Milk (cat #232100, BD Life Science, Franklin Lakes, NJ, USA), followed by overnight incubation at 4 °C with the desired primary antibodies. Blots were washed three times and incubated for 1 hour at room temperature with HRP-conjugated secondary antibodies. Immunoreactive bands were visualized by the ECL method according to standard procedures. The intensity of the immunoreactive bands was quantified using ImageJ software.

### 2.8 siRNA knockdown

LAD-2 cells were seeded in six-well plates at a density of 0.5 × 10^6^ cells/well. Cells were then transfected using 12.5 µl Lipofectamine™ 2000 (Thermofisher, #11668019) and 50 nM of ON-TARGETplus SMARTpool human LRRK1 siRNA or non-targeting control pool siRNA, purchased from Horizon Discovery. Cells were then cultured for additional 72 hours and either left untreated or triggered for 2 minutes with 10 μM of substance P with or without prior treatment with the desired inhibitors. Cells were washed three times with PBS, lysed in lysis buffer and processed for western blotting.

### 2.9 Transient transfection of RBL cells

Transient transfection of RBL cells was performed as previously described (22). Briefly, RBL cells (1.5 x 10^7^) were transfected with a total of 30-60 μg of cDNAs by electroporation at 300V for 20 msec using an ECM 830 electroporator (BTX, USA). The cells were immediately replated in 24-well tissue culture dishes containing growth medium and analyzed after 24 hours.

### 2.10 Immunostaining and confocal image analyses

Immunostaining and confocal analyses were performed as previously described (22). Briefly, cells were grown on 12-mm round glass coverslips (thick #1; Thermo Scientific, Germany), washed three times with PBS and fixed for 20 minutes at room temperature with 4% paraformaldehyde in PBS. Cells were then permeabilized for 20 minutes at room temperature with 0.1% Triton X-100, 5% FBS, and 2% BSA diluted in PBS. Cells were subsequently incubated for 1 hour at room temperature with the primary antibodies, followed by three washes and 1 hour incubation with the appropriate secondary antibodies. After washing, cells were mounted (Fluoromount Aqueous Mounting Medium, cat #F4680, Sigma-Aldrich) and analyzed using a LEICA SP8 STED high resolution laser scanning confocal microscope (Leica, Wetzlar, Germany) using a 63 oil/1.4 numerical aperture objective for imaging. Colocalization analysis of immunostained T7-tagged RILP with EGFP-Rab12 was quantified by calculating the Mander’s overlap coefficients using the ImageJ software.

### 2.11 Statistics and reproducibility

Statistics were performed by using GraphPad Prism Version 9.5.1 (GraphPad Software, CA, USA). The normality of the data was determined using the Shapiro-Wilk test for all groups. Statistical significances were assessed by one-sample t test (hypothetical value of 0), by an unpaired two-tailed Student’s t test or by one-way analysis of variance (ANOVA), followed by Tukey’s test, for multiple comparisons: * p.v < 0.05, ** p.v < 0.01, *** p.v < 0.001, **** p.v < 0.0001. Plots were generated using GraphPad Prism (9.5.1). The results are presented as the mean ± standard error of mean (SEM). The number of replicates is indicated in the figure legends.

## 3 Results

### 3.1 Rab12 is phosphorylated in activated MCs

**Figure 1:**
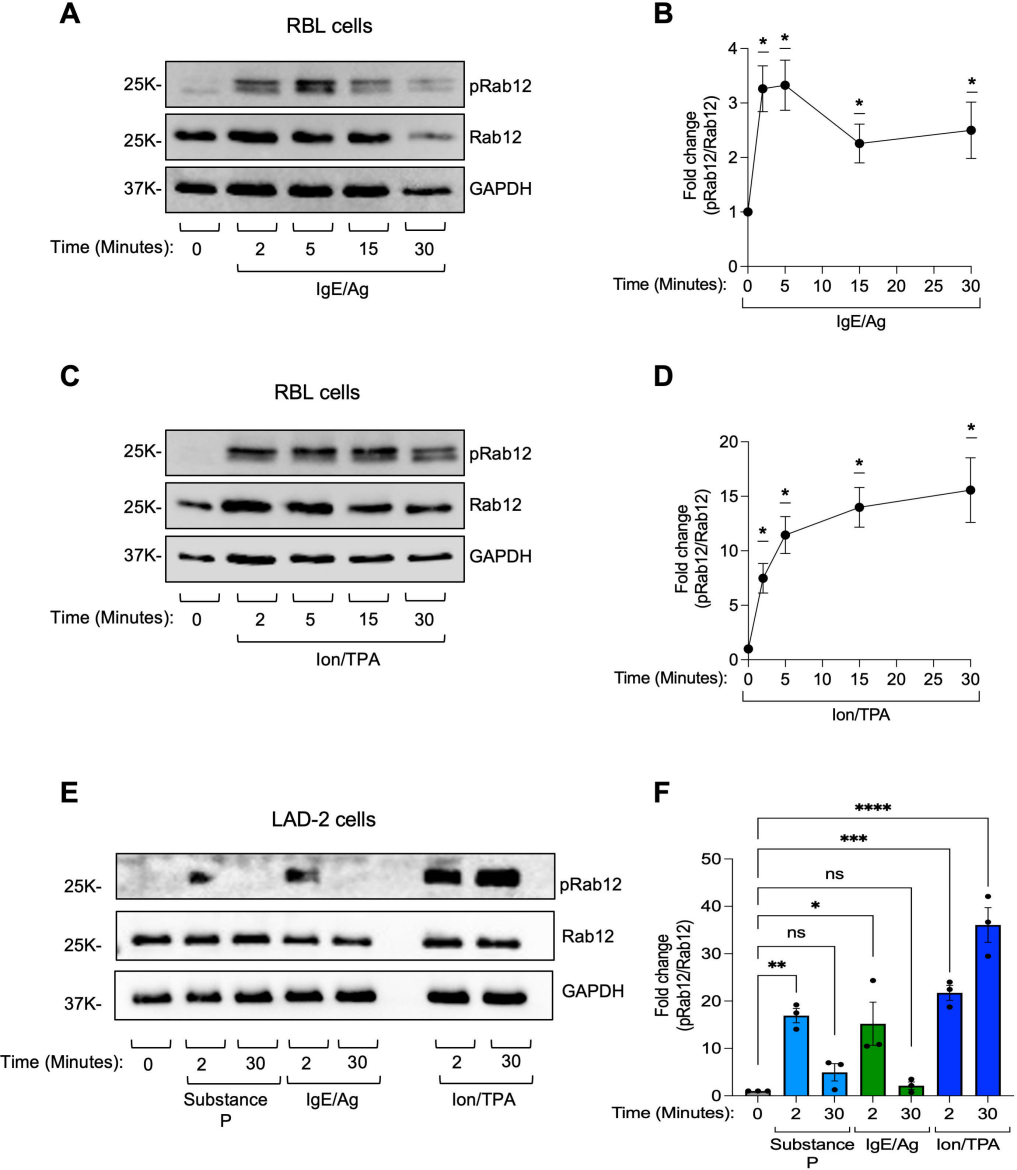
Rab12 phosphorylation is stimulated in activated MCs. RBL cells **(A-D)**, or LAD-2 cells **(E, F)** were either sensitized with IgE **(A, B, E, F)** and subsequently left untreated or treated with antigen, DNP-HSA (IgE/Ag) **(A, B)** or anti IgE (**E, F**), or incubated with a combination of calcium ionophore and TPA (Ion/TPA) (**C-F**), or triggered with substance P (**E, F**), for the indicated time periods. Cell lysates were analyzed by immunoblotting using anti phosphoRab12 antibodies followed by re-probing with anti Rab12 and anti GAPDH antibodies. Representative blots are shown. Blots were quantified by ImageJ software. Data is presented as the ratio between phosphoRab12 to Rab12 normalized to the values of untreated cells. Results are the mean ± SEM derived from 3 independent experiments. *p.v<0.05, **p.v<0.01, ***p.v<0.001, ****p.v<0.0001, assessed by one-sample t-test **(B, D)** or one-way ANOVA **(F)**. ns, not significant.

We first asked whether Rab12 is phosphorylated in MCs. To this end, we used RBL-2H3 cells (hereafter referred to as RBL), a rat mast cell line widely employed as an MC model (34) and shown to regulate secretion through Rab12 (21, 22, 35). Our analysis revealed that Rab12 undergoes phosphorylation in an FcεRI-dependent manner by the addition of the antigen (Ag) DNP-HSA to cells sensitized with DNP-specific IgE (**Figures 1A, B**). Phosphorylation was transient, peaking at 2–5 minutes after cell trigger and declining thereafter (**Figures 1A, B**). Rab12 phosphorylation was also stimulated by the combination of Ion/TPA, though unlike the transient nature of FcεRI-mediated phosphorylation, phosphorylation induced by Ion/TPA was stronger and sustained (**Figures 1C, D**), suggesting the potential involvement of classical PKC in this response. Since Rab12 also regulates MRGPRX2-mediated secretion (35), we also analyzed Rab12 phosphorylation in cells triggered to secretion by substance P, which activates this pathway. RBL cells do not endogenously express Mrgprs (36). We therefore switched to LAD-2 cells, human MCs that endogenously express MRGPRX2, for this purpose. Similarly to the results obtained in RBL cells, both IgE/Ag and Ion/TPA induced Rab12 phosphorylation also in LAD-2 cells, and phosphorylation by IgE/Ag was transient and phosphorylation by Ion/TPA stronger and persistent (**Figures 1E, F**). Rab12 was additionally phosphorylated in response to substance P, and like FcεRI-mediated phosphorylation, phosphorylation stimulated by substance P was transient (**Figures 1E, F**).

### 3.2 Rab12 is phosphorylated in a LRRK2-independent manner

**Figure 2:**
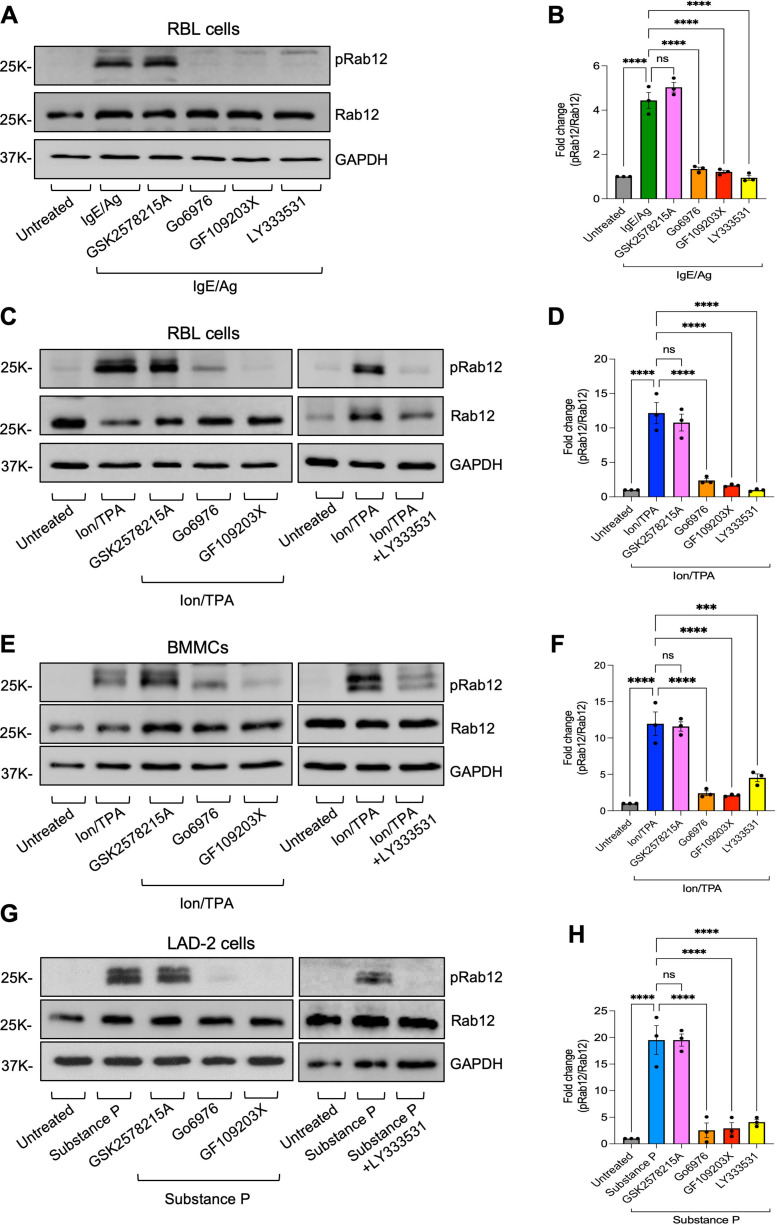
Rab12 phosphorylation is sensitive to inhibitors of PKC. RBL cells **(A-D)**, or BMMCs **(E, F)** or LAD-2 cells **(G, H)**, were either sensitized with IgE and subsequently left untreated or incubated with either GSK2578215A, Go6976, GF109203X or LY333531, as indicated, followed by cell trigger with antigen (DNP-HSA) for 5 minutes **(A, B)**, or Ion/TPA for 30 minutes **(C-F)**, or substance P for 2 minutes **(G, H)**. Cell lysates were analyzed by immunoblotting using anti phosphoRab12 antibodies followed by re-probing with anti Rab12 and anti GAPDH antibodies. Representative blots are shown. Blots were quantified by ImageJ software. Data is presented as the ratio between phosphoRab12 to Rab12 and normalized to untreated cells. Results are the mean ± SEM derived from 3 independent experiments. ***p.v<0.001, ****p.v<0.0001, assessed by one-way ANOVA. ns, not significant.

Unexpectedly, Rab12 phosphorylation by either trigger or in any MC type was insensitive to the LRRK2 inhibitor GSK2578215A (**Figures 2A-H**). In contrast, Rab12 phosphorylation induced by either trigger was significantly inhibited by GF109203X, a general inhibitor of PKCs, Go6976, an inhibitor of classical Ca^2+^-dependent PKCs, or LY333531, that specifically targets the PKCβ1 and PKCβ2 isoforms of Ca^2+^-dependent PKCs (**Figures 2A-H**). We validated these results by analyzing Rab12 phosphorylation also in primary MCs, which were differentiated *in vitro* from mouse bone marrow derived progenitor cells (BMMCs) (**Figures 2E, F**). These results have raised the possibility that a kinase other than LRRK2 may phosphorylate Rab12 in activated MCs.

**Figure 3:**
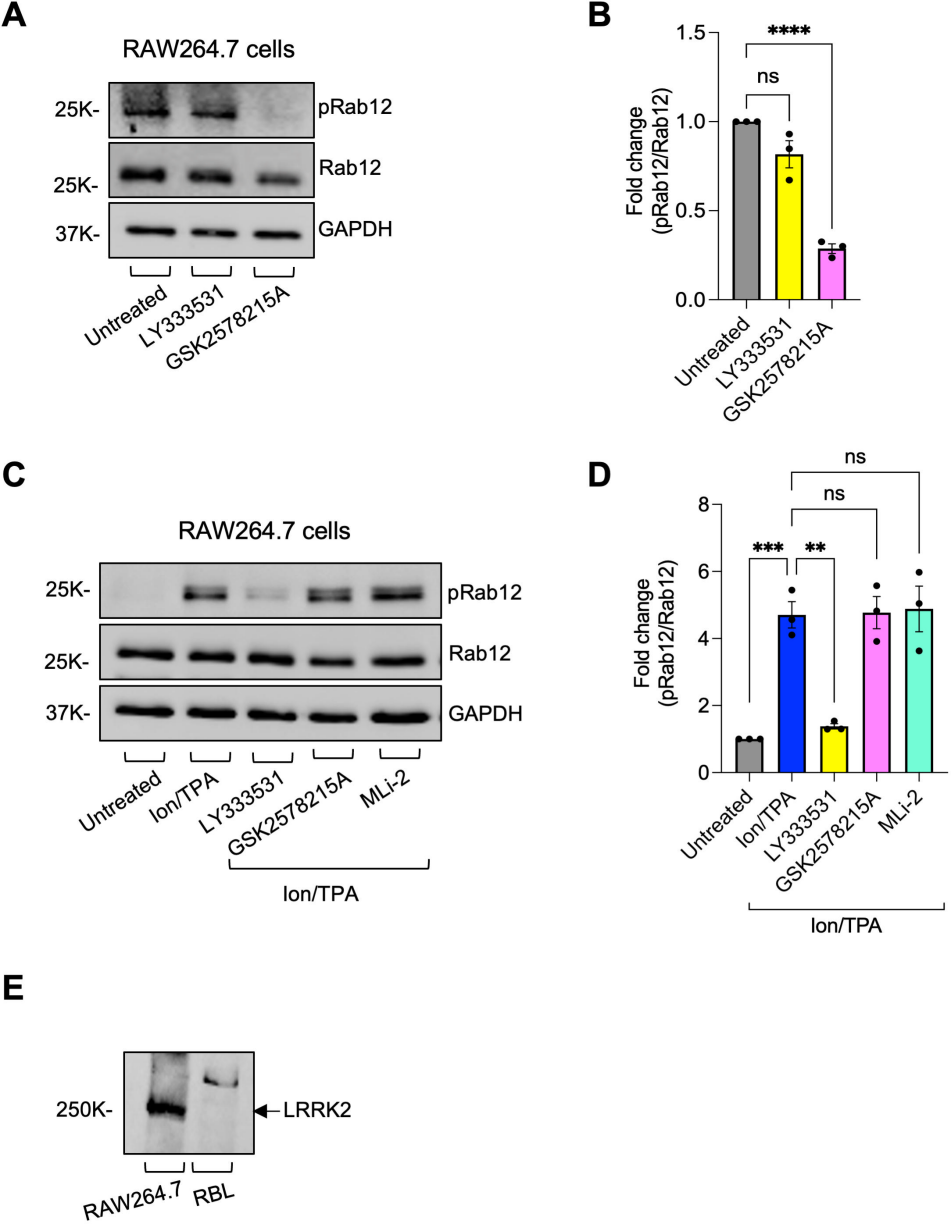
LRRK2 mediates Rab12 phosphorylation in macrophages under basal conditions, whereas a PKC−mediated mechanism is responsible for Rab12 phosphorylation in Ion/TPA-activated cells. RAW264.7 macrophages were either left untreated or incubated with either LY333531 or GSK2578215A inhibitors **(A, B)** or additionally with MLi-2 inhibitor and further triggered for 30 minutes with Ion/TPA **(C, D)**. Cell lysates were analyzed by immunoblotting using anti phosphoRab12 antibodies followed by re-probing with anti Rab12 and anti GAPDH antibodies. Representative blots are shown. Blots were quantified by ImageJ software. Data is presented as the ratio between phosphoRab12 to Rab12 and normalized to untreated cells. Results are the mean ± SEM derived from 3 independent experiments. Cell lysates derived from RAW264.7 macrophages or RBL cells were analyzed by immunoblotting using anti LRRK2 antibodies **(E)**. **p.v<0.01, ***p.v<0.001, ****p.v<0.0001, assessed by one-way ANOVA. Note that the blots in panels **(A, C)** were exposed for different durations [long exposure in **(A)** and short exposure in **(C)**], which explains why basal phosphorylation is not visible in **(C)**. ns, not significant.

To substantiate these results, we analyzed Rab12 phosphorylation in RAW264.7 macrophages, the functions of which are known to be regulated by LRRK2 (37, 38). In these cells, unlike the MCs, Rab12 phosphorylation was already detected under basal conditions (**Figures 3A, B**). Furthermore, phosphorylation was significantly inhibited by GSK2578215A, while it was resistant to LY333531 (**Figures 3A, B**), confirming Rab12 phosphorylation in RAW264.7 macrophages by LRRK2. To investigate if the alternative, LRRK2-independent and PKC-dependent mechanism of Rab12 phosphorylation also operates in RAW264.7 macrophages alongside Rab12 phosphorylation by LRRK2, we investigated the impact of Ion/TPA on Rab12 phosphorylation in these cells. Indeed, Ion/TPA significantly increased Rab12 phosphorylation above the basal phosphorylation (**Figures 3C, D**). Moreover, unlike the basal phosphorylation, which was sensitive to GSK2578215A, Ion/TPA-induced phosphorylation was resistant to GSK2578215A, as well as to MLi-2, a different inhibitor of LRRK2, while it was completely inhibited by LY333531 (**Figures 3C, D**). These findings indicate that Rab12 is phosphorylated by LRRK2 in a cell type-dependent manner. However, it can also be phosphorylated via a PKC-dependent mechanism, independently of LRRK2. Of note, while LRRK2 was clearly detected by immunoblotting RAW264.7 cell lysates, it was below the antibody detection threshold in MCs (**Figure 3E**). Therefore, the cell type dependence of Rab12 phosphorylation by LRRK2 may correlate with the cellular expression level of LRRK2.

### 3.3 LRRK1 phosphorylates both Rab7 and Rab12 in activated MCs

**Figure 4:**
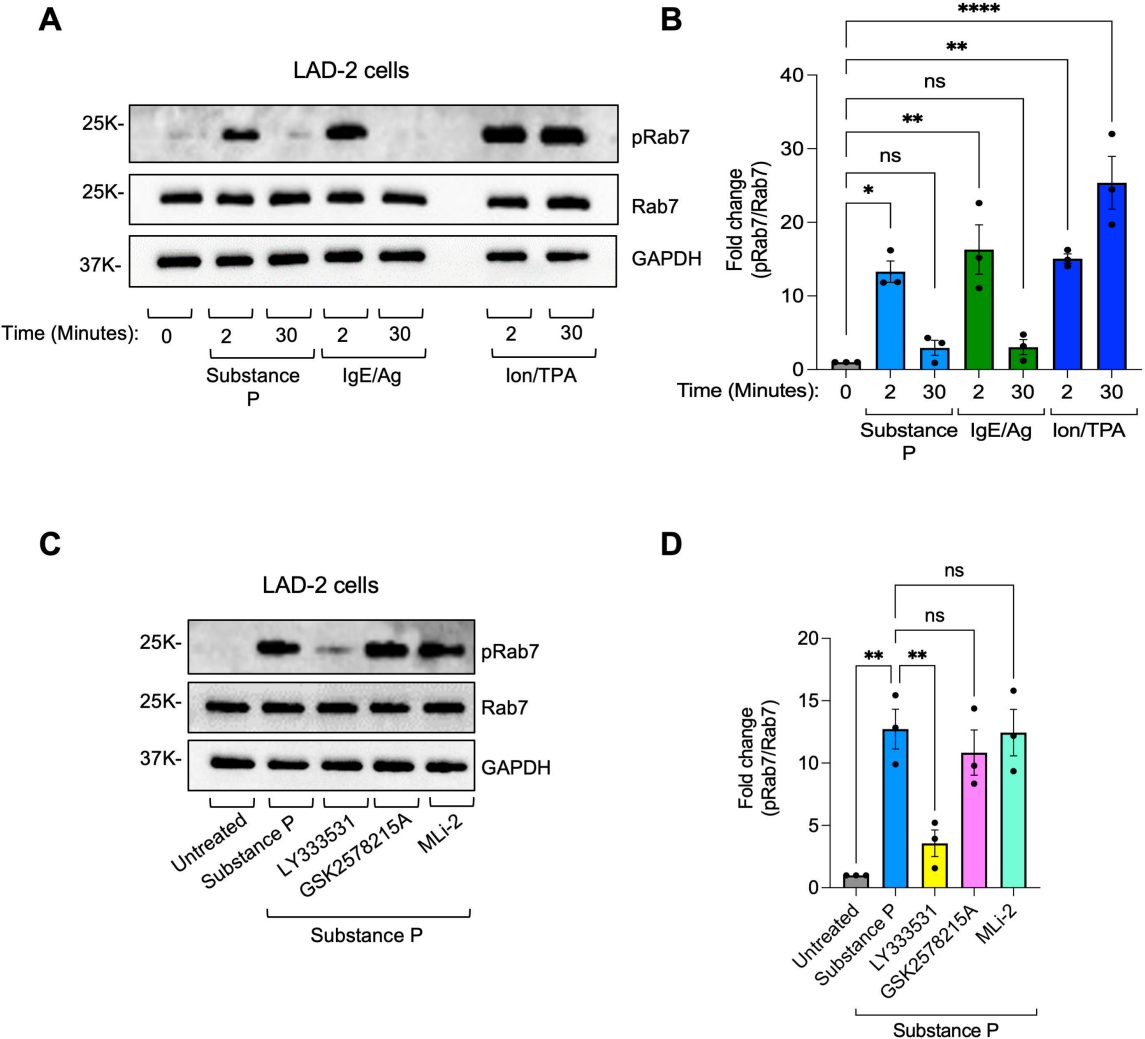
Rab7 is phosphorylated in activated MCs. LAD-2 cells were either left untreated or treated with IgE/Ag, substance P or Ion/TPA for 2 or 30 minutes, as indicated **(A, B)**, or pre-incubated with LY333531, or GSK2578215A or MLi-2 prior to 2 minutes of cell trigger with substance P **(C**, **D)**. Cell lysates were analyzed by immunoblotting using anti phosphoRab7 antibodies followed by re-probing with anti Rab7 antibodies and anti GAPDH antibodies. Representative blots are shown. Blots were quantified by ImageJ software. Data is presented as the ratio between phosphoRab7 to Rab7 and normalized to untreated cells. Results are the mean ± SEM derived from 3 independent experiments. *p.v<0.05, **p.v<0.01, ****p.v<0.0001, assessed by one-way ANOVA. ns, not significant.

PKC was shown to activate the LRRK2 homolog kinase LRRK1 (39). We therefore hypothesized that LRRK1 may replace LRRK2 in phosphorylating Rab12 in PKC-activated cells. To test our hypothesis, we first asked if Rab7, a known substrate of LRRK1 (40), is likewise phosphorylated in activated MCs. Indeed, cell activation by either IgE/Ag, substance P, or Ion/TPA was associated with Rab7 phosphorylation, whereby phosphorylation by either IgE/Ag or substance P was transient, whereas Ion/TPA-induced phosphorylation was sustained (**Figures 4A, B**). Furthermore, Rab7 phosphorylation was resistant to LRRK2 inhibition by GSK2578215A and sensitive to inhibition of PKC by LY333531 (**Figures 4C, D**).

**Figure 5:**
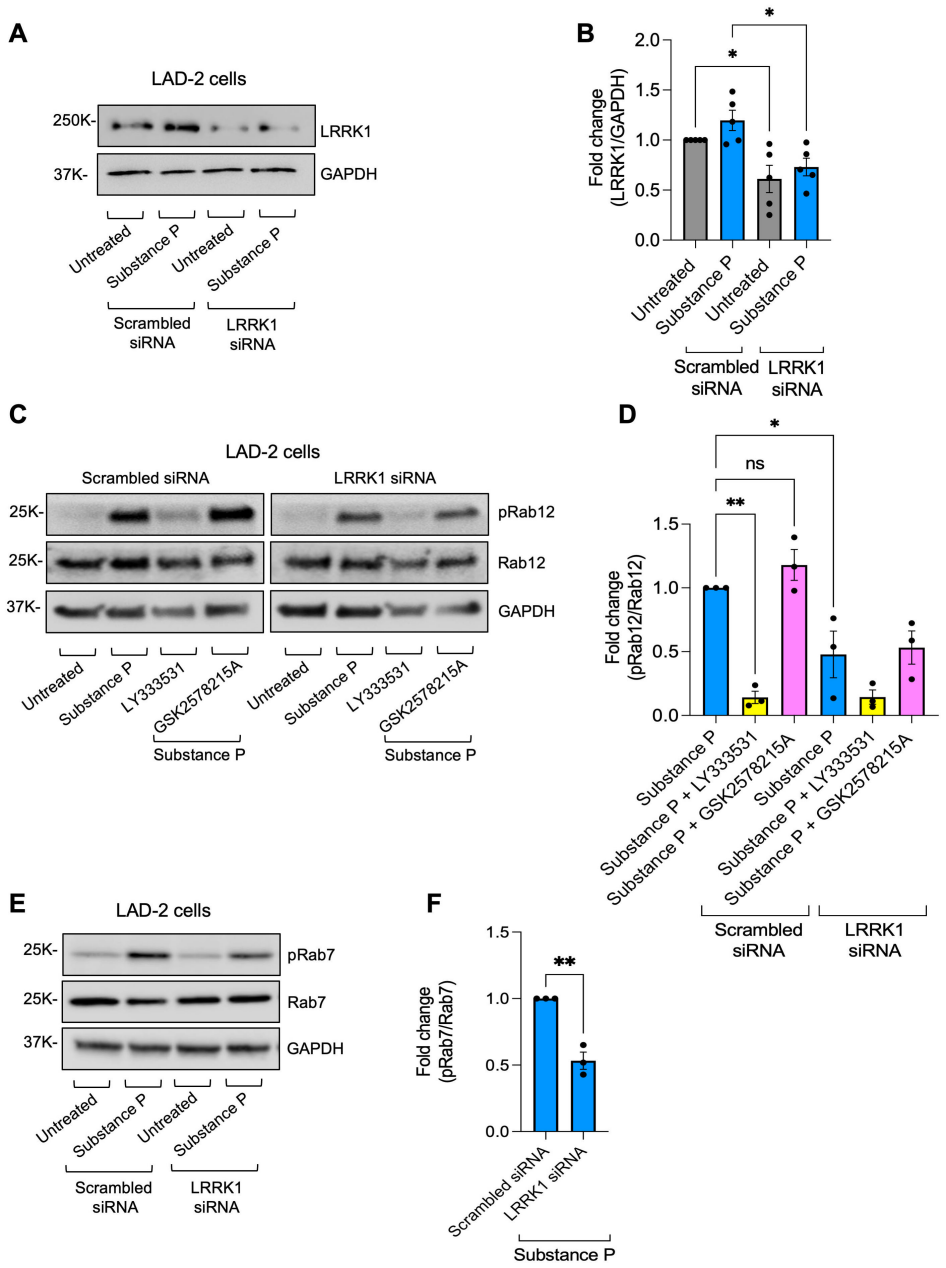
LRRK1 mediates Rab7 and Rab12 phosphorylation in activated MCs. LAD-2 cells were transfected with scrambled, non-targeting control siRNA or LRRK1 targeting siRNA, as indicated **(A-F)**. Cells were then left untreated or triggered for 2 minutes with substance P, in the absence or presence of LY333531 or GSK2578215A, as indicated **(A-F)**. Cell lysates were analyzed by immunoblotting using anti LRRK1 antibodies **(A, B)**, or anti phosphoRab12 antibodies, followed by re-probing with anti Rab12 antibodies and anti GAPDH antibodies **(C, D)**, or anti phosphoRab7 antibodies, followed by re-probing with anti Rab7 and anti GAPDH antibodies **(E, F)**. Representative blots are shown. Blots were quantified by ImageJ software. Data are presented as the ratio of LRRK1 to GAPDH normalized to untreated cells transfected with scrambled non-targeting siRNA **(B)** or the ratio of phosphoRab12 to Rab12 or phosphoRab7 to Rab7, normalized to substance P-activated cells transfected with scrambled non-targeting siRNA, respectively **(D, F)**. Results are mean ± SEM derived from 3–5 independent experiments. *p.v<0.05, **p.v<0.01, assessed by one-way ANOVA **(B, D)** or unpaired two-tailed Student’s t test **(F)**. ns, not significant.

To gain direct proof to the involvement of LRRK1 in the phosphorylation of Rab12, we examined the effect of LRRK1 knockdown on substance P-induced phosphorylation of Rab12, as compared with its impact on phosphorylation of Rab7. We used the human LRRK1-targeting siRNA, which was previously shown to effectively block LRRK1-mediated phosphorylation of Rab7 (41). Although LRRK1 expression was only partially (i.e. by approximately 40%) reduced (**Figures 5A, B**), Rab12 phosphorylation was significantly (i.e. by approximately 50%) inhibited (**Figures 5C, D**). Moreover, Rab12 phosphorylation was reduced to the same extent as phosphorylation of Rab7 (**Figures 5E, F**) and the residual phosphorylation could be further suppressed by LY333531, but not by GSK2578215A (**Figures 5C, D**). These findings therefore implicate LRRK1 in mediating the phosphorylation of both Rab7 and Rab12 in activated MCs.

### 3.4 Phosphorylation by either LRRK1 or LRRK2 differentially affects Rab12 binding to RILP family effectors.

**Figure 6:**
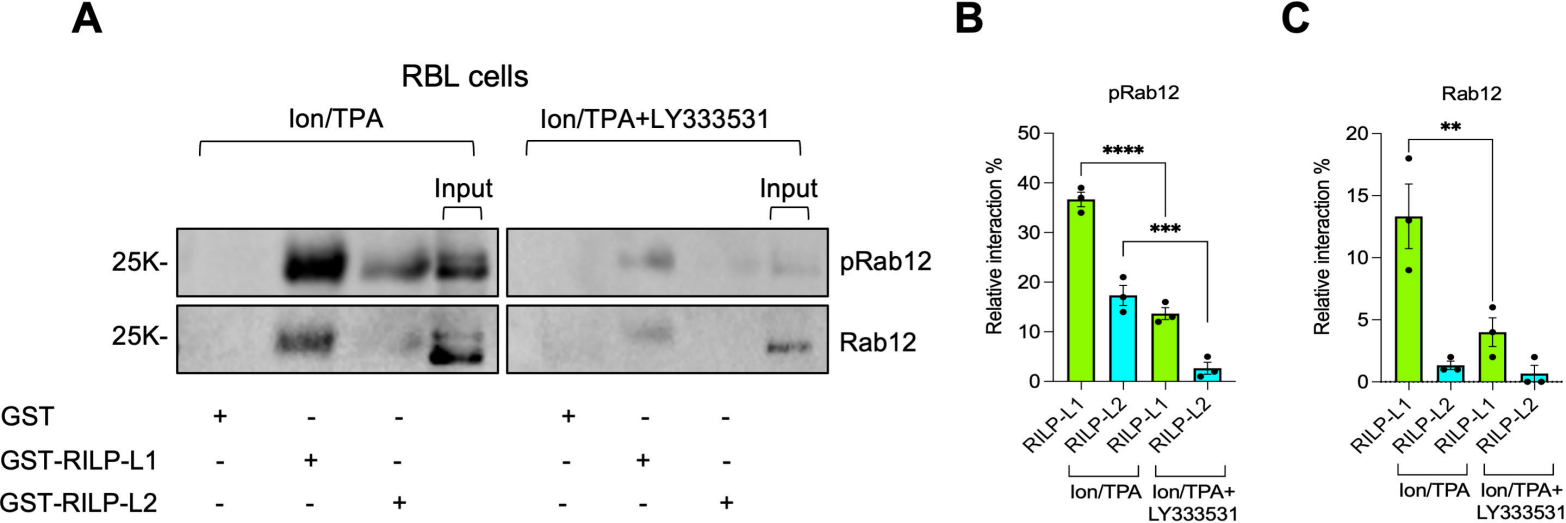
LRRK1-mediated phosphorylation of Rab12 increases its binding affinity for RILP-L1 and RILP-L2. RBL cells were treated with Ion/TPA in the absence or presence of LY333531, as indicated **(A-C).** Cell lysates were subjected to pulldown assays by GST-RILP, GST-RILP-L1 or GST-RILP-L2, immobilized on glutathione agarose beads, as indicated. Bound proteins were resolved by SDS-PAGE and analyzed by immunoblotting using anti phosphoRab12 antibodies followed by re-probing with anti Rab12 antibodies. A representative blot is shown. Input=10% of total protein. Blots were quantified by ImageJ software and the binding of phosphoRab12 **(B)** or total Rab12 **(C)** are presented as percentage of total input. Results are the mean ± SEM derived from 3 independent experiments. **p.v<0.01, ***p.v<0.001, ****p.v<0.0001, assessed by one-way ANOVA.

Since phosphorylation by LRRK2 was shown to enhance Rab12 binding affinity for RILP-L1 and RILP-L2 (25–27), we investigated whether phosphorylation by LRRK1 exerts a similar effect. To this end, we compared the pulldown of Rab12 by RILP-L1 and RILP-L2 from lysates of Ion/TPA-activated MCs, with or without prior treatment with LY333531 to inhibit LRRK1-dependent phosphorylation. RILP-L1 pulled down 38% of the total phosphoRab12 present in Ion/TPA-activated cells and 14% of the total protein (**Figures 6A-C**). RILP-L2 pulled down 18% of phosphorylated Rab12 and only 4% of the total protein (**Figures 6A-C**). Treatment with LY333531 reduced the cellular amount of phosphoRab12 and the amounts of phosphoprotein pulled down by either RILP-L1 or RILP-L2 (**Figures 6A, B**). LY333531 also reduced the pulldown of total Rab12 by RILP-L1 by 60% and by RILP-L2 by 90% (**Figures 6A, C**), revealing their preferable binding to phosphoRab12, with a greater dependence on phosphorylation of RILP-L2, as compared with RILP-L1. These findings demonstrate that similar to phosphorylation by LRRK2, phosphorylation by LRRK1 enhances Rab12 binding affinity for RILP-L1 and RILP-L2.

**Figure 7:**
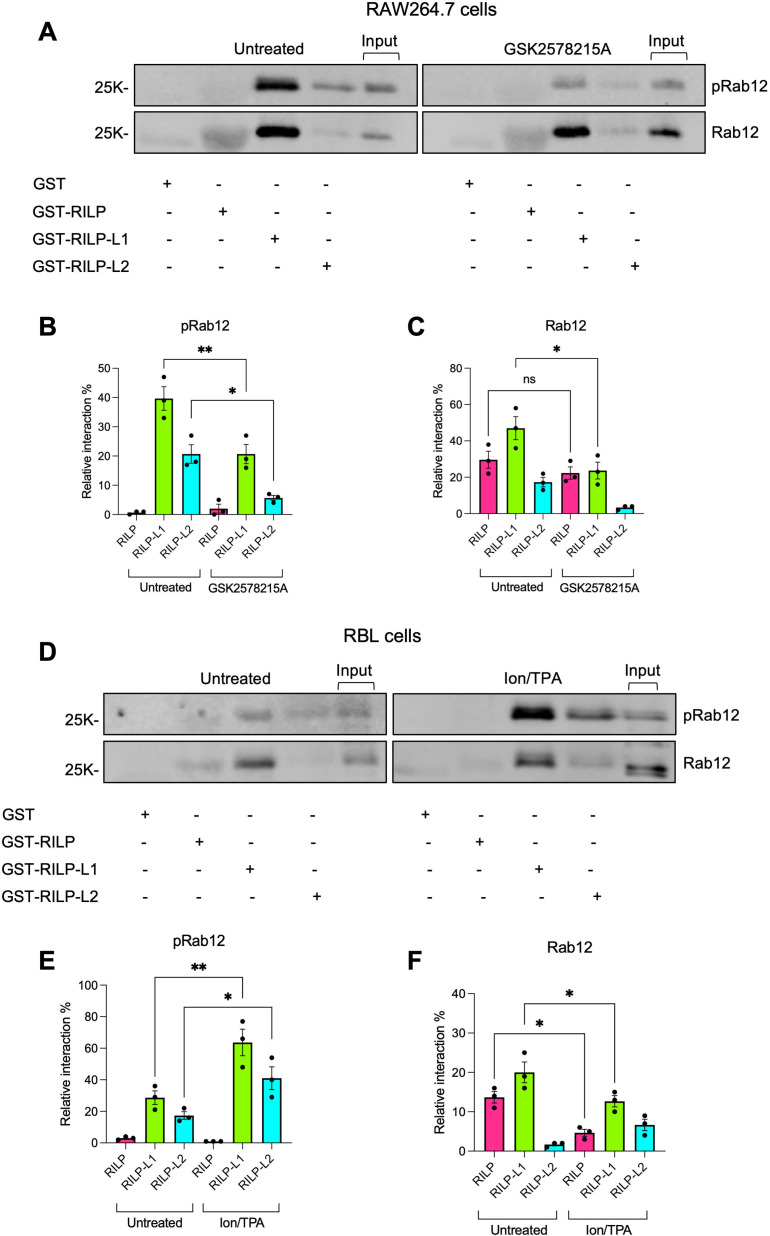
Phosphorylation differentially affects Rab12-RILP family effector binding. RAW264.7 macrophages were either left untreated or incubated with GSK2578215A, as indicated **(A-C).** RBL cells were either left untreated or triggered with Ion/TPA, as indicated **(D-F)**. Cell lysates were subjected to pulldown assays by GST-RILP, GST-RILP-L1 or GST-RILP-L2, immobilized on glutathione agarose beads, as indicated. Bound proteins were resolved by SDS-PAGE and analyzed by immunoblotting using anti phosphoRab12 antibodies **(B, E)** followed by re-probing with anti Rab12 antibodies **(C, F)**. Representative blots are shown. Input=10% of total protein. Blots were quantified by ImageJ software and the binding of phosphoRab12 **(B, E)** or total Rab12 **(C, F)** are presented as percentage of total input. Results are the mean ± SEM derived from 3 independent experiments. *p.v<0.05, **p.v<0.01, assessed by one-way ANOVA. ns, not significant.

**Figure 8:**
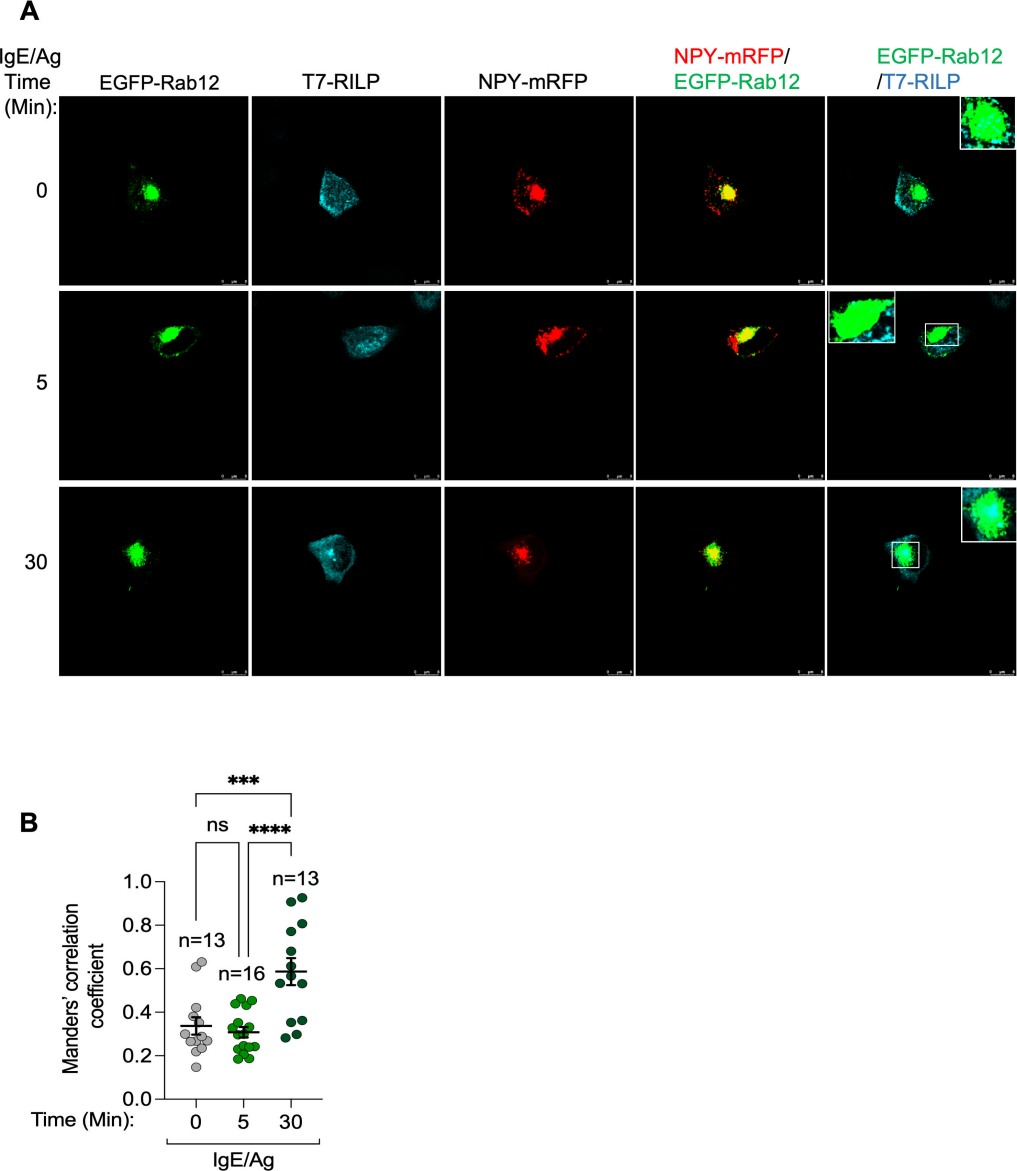
Cell trigger enhances Rab12-RILP interaction in a time-dependent manner. **(A)** RBL cells were transiently co-transfected with 15 μg of plasmid encoding NPY-mRFP (red), 15 μg of pEGFP-C1-Rab12 (green) and 20 μg of pEF-T7-RILP and grown for 24 h in the presence of IgE (1:500 dilution). Cells were then left untreated (UT) or activated by 50 ng/ml of DNP-HSA (IgE/Ag) for the indicated time periods. Cells were subsequently fixed and immunostained with monoclonal antibodies directed against T7, followed by Hilyte Plus 647-conjugated goat anti-mouse IgG (cyan). Cells were visualized by confocal microscopy. n = 2 independent experiments. Bar = 8 μm. The Insets are the enlargements of the boxed areas. **(B)** Manders’ overlap coefficient for immunostained T7-tagged RILP and EGFP-Rab12 was determined by calculating the fraction of green pixels (Rab12 signal) that overlap with cyan pixels (RILP signal). Results are the mean ± SEM. ***p.v<0.001, ****p.v<0.0001, assessed by one-way ANOVA. Numbers indicate total number of analyzed cells. ns, not significant.

**Figure 9:**
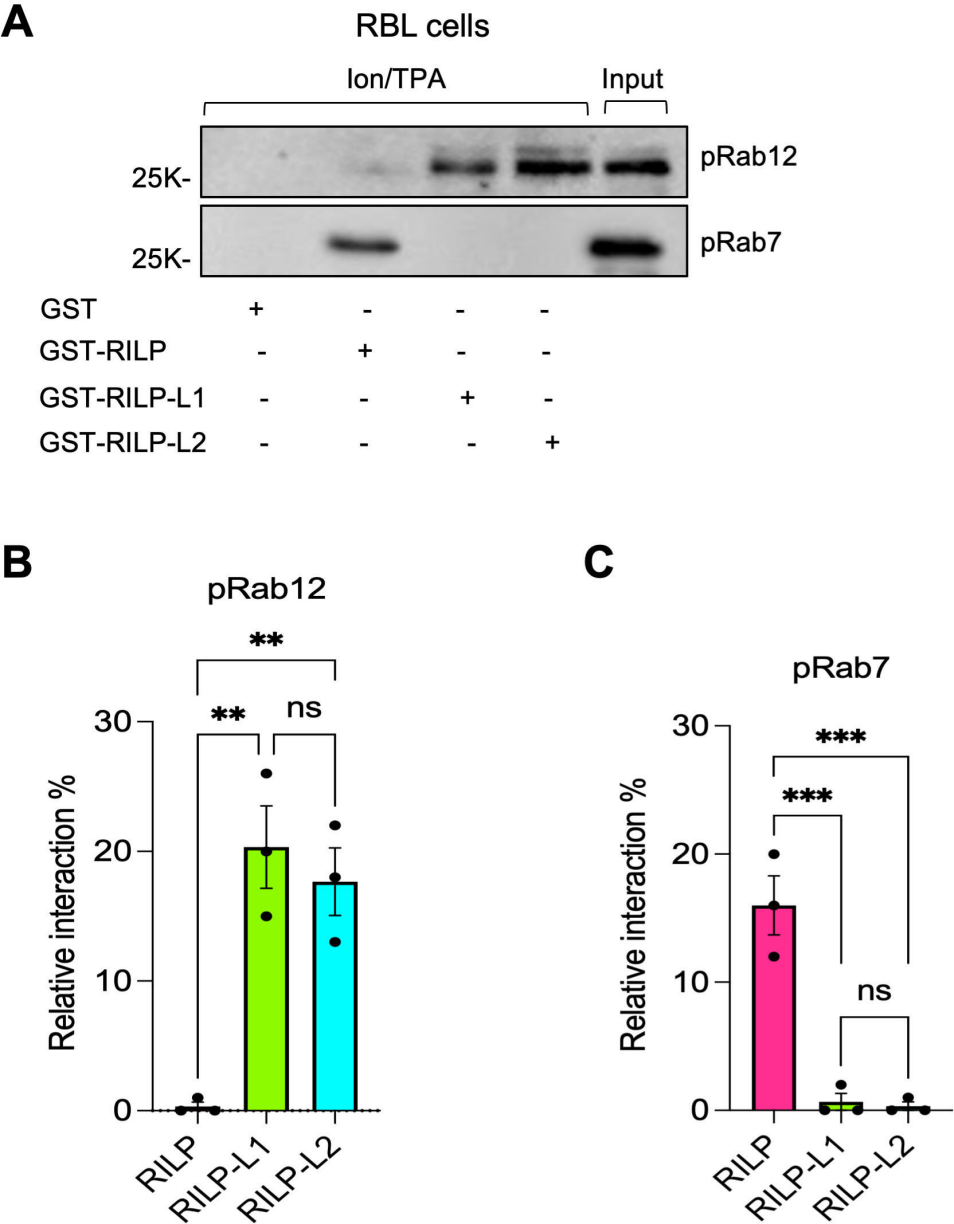
Phosphorylation has opposite effects on Rab12 versus Rab7 binding affinity for RILP. Lysates derived from Ion/TPA-activated RBL cells were subjected to pulldown assays by GST-RILP, GST-RILP-L1 or GST-RILP-L2, immobilized on glutathione agarose beads, as indicated **(A-C)**. Bound proteins were resolved by SDS-PAGE and analyzed by immunoblotting using anti phosphoRab12 antibodies **(B)** followed by re-probing with anti phosphoRab7 antibodies **(C)**. Representative blots are shown. Input=10% of total protein. Blots were quantified by ImageJ software. Binding of phosphoRab12 **(B)** and phosphoRab7 **(C)** is presented as % of total input. Results are the mean ± SEM derived from three independent experiments. **p.v<0.01, ***p.v<0.001, assessed by one-way ANOVA. ns, not significant.

We also investigated how phosphorylation by either LRRK1 or LRRK2 impacts Rab12 interaction with RILP, which has not been studied before. Pulldown assays were performed using lysates derived from either RAW264.7 macrophages, where Rab12 is phosphorylated by LRRK2 under basal conditions, in the absence and presence of GSK2578215A, to inhibit phosphorylation by LRRK2, or from untreated RBL cells, in which Rab12 is non-phosphorylated, or Ion/TPA-activated RBL cells, in which Rab12 is robustly phosphorylated by LRRK1. All three RILP family members pulled down Rab12 from RAW264.7 cell lysates and in agreement with the previous reports (26, 42), both RILP-L1 and RILP-L2 effectively pulled down the LRRK2-phosphorylated Rab12 (**Figures 7A, B**). However, in sharp contrast, RILP exclusively pulled down the non-phosphorylated protein (**Figures 7A, C**). Furthermore, while GSK2578215A reduced by 50% the pulldown of Rab12 by RILP-L1 and by 80% its pulldown by RILP-L2 (**Figure 7C**), GSK2578215A had no significant effect on Rab12 pulldown by RILP (**Figure 7C**). Similarly, unlike RILP-L1 and RILP-L2, which pulled down phosphoRab12 from Ion/TPA-activated MCs (**Figures 7D, E**), RILP exclusively pulled down the non-phosphorylated protein (**Figures 7D-F**). Furthermore, RILP pulled down significantly more Rab12 from lysates of untreated cells, where Rab12 is predominantly non-phosphorylated, than from lysates of Ion/TPA-treated cells (**Figure 7F**). These results suggest that under physiological stimulation, such as IgE/Ag activation, Rab12 interacts with RILP at later stages of stimulation, when Rab12 phosphorylation declines. To test this prediction, we analyzed the colocalization of Rab12 and RILP in resting RBL cells and in cells stimulated with IgE/Ag for 5 minutes, when Rab12 is phosphorylated, and for 30 minutes, when phosphorylation wanes. Because anti-Rab12 antibodies are not suitable for imaging-based analyses, we co-transfected cells with EGFP-Rab12 and T7-tagged RILP. Co-expression of the SG marker NPY-mRFP enabled visualization of SG distribution under these conditions. Consistent with our previous results (26), in resting cells Rab12 localized to the perinuclear region, whereas the SGs were distributed between the perinuclear area, where they colocalized with Rab12, and the cell periphery (**Figure 8A**). Under these conditions, RILP was mostly cytosolic, though a small fraction colocalized with Rab12 at the perinuclear region (**Figures 8A, B**). Short physiological stimulation with antigen (i.e. for 5 minutes) had little effect on the distribution of Rab12, but it reduced, though not significantly, the fraction of Rab12 that colocalized with RILP (**Figures 8A, B**). The SGs remained distributed between the perinuclear region and the cell periphery, where they appeared to cluster (**Figure 8A**), consistent with SG fusion that occurs during antigen-triggered compound exocytosis (43, 44). In sharp contrast, prolonged antigen stimulation (i.e. for 30 minutes), led to a significant increase in the colocalization of Rab12 with RILP (**Figures 8A, B**) and exclusive accumulation of the SGs at the perinuclear region (**Figure 8A**). These findings support a dynamic interaction between Rab12 and RILP that strengthens during sustained activation, consistent with our previous demonstration of Rab12 activation in response to cell triggering (22). However, this interaction depends on Rab12 dephosphorylation and likely contributes to SG retention in stimulated cells. Taken together, our results indicate that phosphorylation by either LRRK2 or LRRK1 has a similar influence on Rab12-effector interactions, increasing Rab12 affinity for RILP-L1, increasing even more its affinity for RILP-L2, but decreasing its affinity for RILP. In sharp contrast, phosphoRab7 was neither pulled down by RILP-L1 nor by RILP-L2 (**Figures 9A-C**). Furthermore, consistent with previous results (40), phosphoRab7 was efficiently pulled down by RILP (**Figures 9A-C**). Therefore, LRRK1-mediated phosphorylation oppositely affects Rab7 versus Rab12 interactions with RILP.

## 4 Discussion

**Figure 10:**
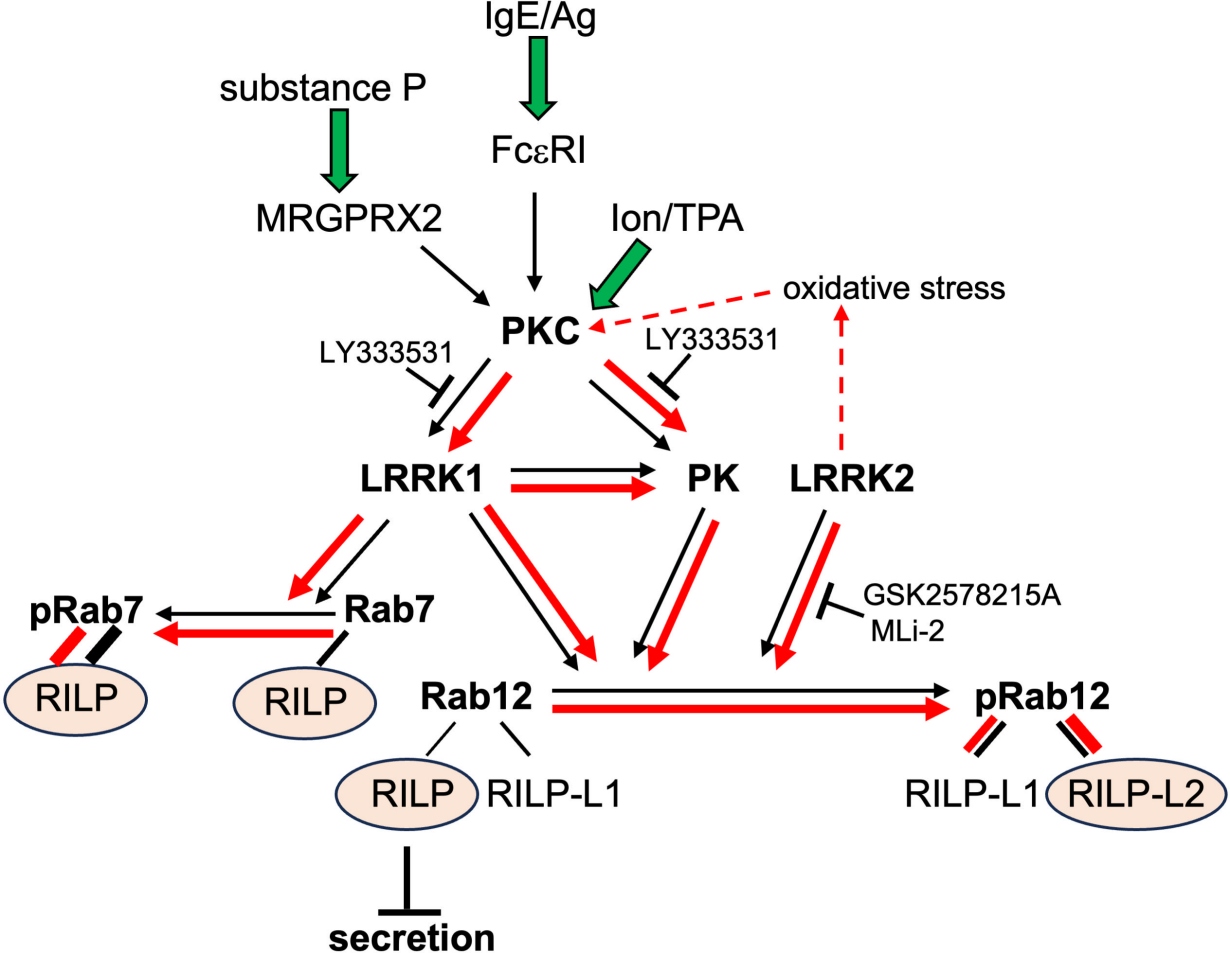
A model depicting the role of phosphorylation in controlling Rab12 interactions with RILP family members. According to our model, in cells with high LRRK2 expression, such as macrophages, or in cells carrying the hyperactive LRRK2-G2019S mutant, Rab12 is phosphorylated by LRRK2 under basal conditions. In contrast, in cells with low LRRK2 levels, such as MCs, Rab12 phosphorylation occurs independently of LRRK2 through a PKC and LRRK1-dependent mechanism in response to cell stimulation. LRRK1 may directly phosphorylate Rab7 and Rab12 or activate another, yet unidentified kinase (PK) that phosphorylates Rab12 and may itself be activated by PKC. Under either condition, non-phosphorylated Rab12 interacts with RILP and RILP-L1, whereas phosphorylated Rab12 (pRab12) preferentially binds RILP-L2. In contrast, phosphorylation of Rab7 increases its affinity for RILP. Activating mutations in LRRK2 (red arrows) shift the equilibrium toward increased pRab12 and enhanced binding to RILP-L2 at the expense of RILP, thereby disturbing cellular homeostasis. Similarly, conditions that hyperactivate PKC, such as oxidative stress during aging or the presence of activating LRRK2 mutations, may lead to excessive LRRK1 activation, producing comparable effects on Rab12–RILP family interactions and on RILP distribution between Rab7 and Rab12. We propose that under physiological conditions, Rab12 phosphorylation is tightly regulated. In MCs, phosphorylation enables trigger-dependent mobilization of the SGs, while dephosphorylation enhances their minus-end transport, thereby limiting secretion. Dysregulation of Rab12 phosphorylation disrupts its interaction balance with RILP family members, contributing to disease progression, such as in PD.

Rab12 is one of the least characterized Rab GTPases. Previous studies have implicated Rab12 in facilitating the transport of the transferrin receptor from the endocytic recycling compartment to lysosomes (32), stimulating autophagy by controlling the transport of the PAT4 amino acid transporter (45), controlling the retrograde transport of the shiga toxin from the plasma membrane to the TGN (46) and the post-Golgi trafficking of the EGF receptor (47), suggesting that Rab12 may execute its multiple functions through interactions with diverse effector proteins. In agreement with this notion, we have identified RILP, as the Rab12 effector that mediates Rab12 negative regulation of MC secretion, by recruiting the motor protein dynein to the SGs and promoting their minus end transport (22). This function could not be recapitulated by RILP-L1 or RILP-L2, Rab12-related effectors (22, 26), supporting the notion that Rab12 mediates its diverse functions through distinct effectors. However, the precise assignment of effector-function has remained by largely unresolved. Proteomic screens of mouse embryonic fibroblasts from a knock-in mouse expressing the hyperactive mutant of LRRK2, LRRK2-G2019S, the most common cause of familial PD (28), or LRRK2-G2019S knock-in cell lines, such as HEK293, have identified Rab12 as a substrate of LRRK2, suggesting its involvement in PD (25, 27). This is consistent with its robust phosphorylation observed in PD model brains (48) and in peripheral blood mononuclear cells derived from LRRK2-G2019S carriers (49). However, aside from A549 lung cancer cells (50) and RAW264.7 macrophages, shown here, both of which highly express LRRK2 (37, 38), and human peripheral mononuclear blood cells, in which LRRK2 expression was stimulated by TPA and interferon-γ (51), little is known about Rab12 phosphorylation in the absence of hyperactive LRRK2 mutant expression. We were prompted to analyze Rab12 phosphorylation in MCs. Because LRRK2-mediated phosphorylation of Rab12 was shown to increase its affinity for its effector proteins RILP-L1 and RILP-L2 (25, 27), and since Rab12 binds all three members of the RILP family (22, 25–27), we reasoned that if phosphorylation differentially modulates Rab12-RILP interactions, it could orchestrate Rab12 distribution among its effectors and thereby regulate its distinct cellular functions. Addressing this question yielded unexpected findings. While we found that Rab12 is phosphorylated in MCs, this phosphorylation is not mediated by LRRK2. This conclusion is supported by several observations. First, unlike Rab12 phosphorylation in macrophages, which occurs under basal conditions, Rab12 phosphorylation in MCs occurs only in activated cells, indicating that the responsible kinase activity depends on receptor signalling. Second, while the basal phosphorylation of Rab12 in macrophages is sensitive to LRRK2 inhibitors consistent with its phosphorylation by endogenous LRRK2, Rab12 phosphorylation in activated MCs is resistant to these inhibitors. Finally, while Rab12 phosphorylation in macrophages is unaffected by PKC inhibition, in MCs it is significantly reduced by PKC inhibitors, particularly by LY333531, which targets PKCβ. These results implicate PKC, and specifically PKCβ, in Rab12 phosphorylation in activated MCs. However, since Rab12 phosphorylation in MCs was detected by the antibodies that are directed against phosphoserine 106 (Ser105 in rodents), the known LRRK2 phosphorylation site, which does not conform to the PKC consensus sequence, we hypothesized that LRRK1, which was previously shown to be activated by PKC (39), may phosphorylate Rab12 in activated MCs. Indeed, we show that Rab7, a known substrate of LRRK1, is phosphorylated in activated MCs, displaying a similar drug sensitivity as Rab12. Furthermore, partial knockdown of LRRK1 significantly reduces the phosphorylation of both Rab7 and Rab12 and to the same extent. These findings demonstrate that while LRRK1 and LRRK2 have a distinct set of substrates, including Rab8 and Rab10 that are exclusively phosphorylated by LRRK2, but not by LRRK1 (41), and Rab7, which is selectively phosphorylated by LRRK1, but not by LRRK2 (40, 41), they may share Rab12 as their common substrate, highlighting its unique status. Furthermore, our results show that phosphorylation of Rab12 by either LRRK1 or LRRK2 similarly affects its interactions with RILP family effectors, consistent with phosphorylation occurring at a common site. However, while phosphorylation by either kinase increases Rab12 interactions with RILP-L1 or RILP-L2, RILP exclusively binds to non-phosphorylated Rab12. These findings indicate that phosphorylation governs the selective distribution of Rab12 among its effectors, shifting the balance of Rab12-containing complexes toward RILP-L1 and RILP-L2 at the expense of the Rab12–RILP complex (see model, **Figure 10**). Furthermore, since unlike the preferential binding of RILP to non-phosphorylated Rab12, RILP displays stronger affinity for phosphorylated Rab7, LRRK1-mediated phosphorylation may also redirect RILP binding from Rab12 to phosphorylated Rab7, further reducing Rab12-RILP complex formation (**Figure 10**). Although the specific role of this kinase in regulating MC function in allergy and neuroinflammation remains to be elucidated, we propose that activation of this kinase, through phosphorylation of Rab12 and Rab7, enables external triggers to coordinate the transport of lysosome-related SGs with that of degradative lysosomes, thereby coupling SG secretion with receptor downregulation.

In conclusion, we identified LRRK1 as a novel player in the signalling pathways elicited by either FcϵRI or MRGPRX2 in MCs, leading to the phosphorylation of Rab7 and Rab12. Our results also have broader implications. We show that Rab12 phosphorylation can be mediated by LRRK2 alone, LRRK1 alone, or by both kinases together, depending on their relative expression levels or activation status. This conclusion aligns with the positive correlation between activating mutations in LRRK2, which favor this kinase over LRRK1, and the progression of PD (28), as well as with the detection of phosphoRab12 under basal conditions only in cells that express high levels of LRRK2. In contrast, only the combined knockout of LRRK1 and LRRK2 results in age-dependent neurodegeneration in an animal model, supporting the notion of synergy between these two kinases under physiological conditions and their joint involvement in sporadic, age-related PD (52–54). Indeed, oxidative stress induced by aging or by LRRK2 activation may activate PKC, which in turn may activate LRRK1. Since phosphorylation by either kinase similarly alters Rab12 affinity for its RILP family effectors, we propose that hyperphosphorylation of Rab12 by either kinase disrupts the balance between Rab12 and Rab7-effector interactions, thereby perturbing cellular homeostasis and contributing to disease progression (see Model, **Figure 10**).

### 4.1 Limitations of the study

In this study, we demonstrate that Rab12, a known physiological substrate of LRRK2, can also be phosphorylated in a LRRK1 and PKC-dependent manner. We show that the choice of kinase is dependent on the cell type or activation trigger. Furthermore, we establish that like LRRK2-mediated phosphorylation, LRRK1-mediated phosphorylation increases Rab12 affinity for RILP-L1 and RILP-L2. However, phosphorylation by either kinase reduces Rab12 affinity for RILP. Despite these findings, several questions remain unresolved. The precise cellular functions of Rab12 are not yet fully understood. We previously identified Rab12 as a regulator of minus-end transport of MC SGs through its interaction with RILP and recruitment of the dynein motor (22). However, we observed no significant change in SG positioning in LRRK1-knockdown cells, likely due to the partial knockdown achieved. In addition, the specific organelles whose transport is regulated by the Rab12-RILP complex in cell types other than MCs remain to be identified. Furthermore, although our results clearly implicate both PKC and LRRK1 in mediating Rab12 phosphorylation, we cannot rule out the involvement of an additional kinase. PKC and LRRK1 might independently activate this third kinase, or PKC could activate LRRK1, which in turn either phosphorylates Rab7 and Rab12 directly or activates another kinase downstream (see Model, **Figure 10**). Future studies addressing these questions will help clarify the broader physiological and pathological significance of Rab12 phosphorylation and its effector interactions in allergy, neurogenic inflammation, and Parkinson’s disease.

## Data Availability

The original contributions presented in the study are included in the article/supplementary material. Further inquiries can be directed to the corresponding author/s.
